# Impact of dose reduction and multi‐parametric harmonization on multi‐organ radiomics robustness in total‐body PET imaging: Methodological implications for quantitative theranostics

**DOI:** 10.1002/mp.70731

**Published:** 2026-10-22

**Authors:** Mohammad Saber Azimi, Maryam Cheraghi, Habib Zaidi

**Affiliations:** ^1^ Doctoral School of Applied Informatics and Applied Mathematics Óbuda University Budapest Hungary; ^2^ University Research and Innovation Center Óbuda University Budapest Hungary; ^3^ PET‐CT Unit Department of Nuclear Medicine Masih Daneshvari Hospital Shahid Beheshti University of Medical Sciences Tehran Iran; ^4^ Division of Nuclear Medicine & Molecular Imaging Geneva University Hospital Geneva Switzerland; ^5^ Department of Nuclear Medicine and Molecular Imaging University of Groningen Groningen Netherlands; ^6^ Department of Nuclear Medicine University of Southern Denmark Odense Denmark

**Keywords:** ComBat harmonization, dose robustness, feature reproducibility, radiomics, total‐body PET

## Abstract

**Background:**

Radiomic features derived from positron emission tomography (PET) are sensitive to count statistics and acquisition protocol variability, thereby limiting quantitative reliability in low‐dose and multi‐center total‐body PET imaging.

**Purpose:**

This study evaluated dose robustness of PET radiomics and assessed the impact of multi‐parameter ComBat harmonization on feature reproducibility.

**Methods:**

A multi‐center total‐body PET dataset comprising 550 patients from two institutions was retrospectively analyzed. Seven dose levels were simulated from list‐mode data (Standard; dose reduction factors (DRF) of 2, 4, 10, 20, 50, and 100). Automatic deep learning–based segmentation was applied. A total of 107 Image Biomarker Standardization Initiative (IBSI)‐compliant radiomic features were extracted from standardized uptake value (SUV)‐normalized images. Feature‐level ComBat harmonization was performed under five configurations (scanner manufacturer, pixel spacing, radiopharmaceutical, scatter correction, and non‐harmonized baseline). Reproducibility was quantified using intraclass correlation coefficient (ICC (2,1)) across dose levels, organs, and harmonization settings.

**Results:**

Dose reduction produced organ‐ and feature‐dependent effects. Overall, 32.7% of the features achieved good or excellent reproducibility (ICC ≥ 0.75) in at least one anatomical region. Selected first‐order and intensity‐associated features retained good reproducibility in several anatomical regions under moderate dose reduction (DRF ≤ 20), whereas severe count reduction (DRF 50–100) was associated with greater organ‐ and feature‐dependent instability, particularly among higher‐order texture features in vascular and lower‐contrast soft‐tissue regions. Harmonization produced organ and feature‐dependent changes but did not uniformly improve cross‐dose reproducibility or compensate for noise‐induced instability.

**Conclusion:**

Radiomics robustness in total‐body PET is strongly influenced by dose level, anatomy, and acquisition variability. Moderate dose reduction preserves selected intensity‐based features, while extreme count reduction compromises higher‐order textures. Harmonization altered reproducibility patterns but did not uniformly improve cross‐dose agreement or fully offset noise‐induced instability, underscoring the need for dose‐aware feature selection in multi‐center PET radiomics.

## 1 INTRODUCTION

Positron emission tomography (PET) plays a central role in oncologic imaging by enabling quantitative assessment of tracer uptake, tumor metabolism, and whole‐body disease burden.^1^, ^2^ The recent emergence of total‐body PET systems has further transformed the field by providing unprecedented sensitivity, extended axial field‐of‐view coverage, and substantially improved count statistics.^3^, ^4^ These technological advances have opened new opportunities for ultra‐low‐dose imaging, longitudinal monitoring, and multi‐organ quantitative analysis while maintaining clinically acceptable image quality.^5^, ^6^

In parallel with hardware developments, PET radiomics has emerged as a powerful approach for extracting high‐dimensional quantitative features that characterize lesion heterogeneity beyond conventional standardized uptake values (SUVs).^7^, ^8^, ^9^ Radiomic features derived from PET images have demonstrated potential in prognostic modeling, treatment response assessment, and precision oncology workflows.^10^, ^11^, ^12^ However, the reliability of these quantitative biomarkers critically depends on their robustness to variations in acquisition and reconstruction conditions, including partial volume effects and correction strategies that may substantially alter quantitative metrics and texture patterns.^13^, ^14^, ^15^

Despite the increased sensitivity of total‐body PET, quantitative PET metrics remain inherently dependent on count statistics.^16^, ^17^ Retrospective dose reduction through temporal subsampling of list‐mode acquisitions, which simulates reduced injected activity or shorter acquisition times, can lead to elevated image noise and altered spatial texture patterns.^18^, ^19^, ^20^, ^21^ Several studies have reported that reduced count levels significantly influence both conventional PET metrics and higher‐order radiomic features, raising concerns about their stability under low‐dose conditions.^22^, ^23^, ^24^, ^25^, ^26^, ^27^ Such variability poses a substantial challenge for longitudinal imaging, dose‐optimization strategies, and quantitative multi‐center studies.

Beyond dose‐related effects, quantitative PET imaging is further affected by non‐biological variability introduced by differences in scanner hardware, voxel size, reconstruction algorithms, and correction techniques across institutions.^28^, ^29^, ^30^, ^31^ These inter‐center discrepancies can substantially alter radiomics feature distributions and hinder the integration of heterogeneous datasets.^32^ Statistical harmonization approaches, such as ComBat‐based feature‐level adjustments, have therefore been proposed to mitigate scanner‐ and protocol‐related variability and improve cross‐center comparability of quantitative imaging biomarkers.^33^ In addition, recent investigations have shown that radiomics reproducibility is strongly influenced by partial volume correction strategies and anatomical region selection, further emphasizing the need for methodological standardization.^34^, ^35^

While the independent effects of dose reduction and multi‐center variability have been investigated in prior studies, their combined impact remains insufficiently characterized.^36^, ^37^ In particular, it is unclear how count reduction interacts with scanner‐ and acquisition‐related heterogeneity, and whether metadata‐guided harmonization strategies can preserve radiomics feature reproducibility under low‐count conditions. The unique sensitivity of total‐body PET systems provides a distinctive opportunity to systematically evaluate radiomics robustness across a broad spectrum of simulated dose levels while maintaining intrinsic spatial co‐registration derived from a single list‐mode acquisition.

We hypothesized that dose reduction significantly alters the reproducibility of total‐body PET radiomic features and that harmonization strategies informed by scanner and acquisition metadata can partially mitigate this variability. Therefore, the aim of this study was to evaluate the robustness of quantitative PET radiomic features across multiple retrospectively simulated dose levels in a multi‐center total‐body PET cohort and to assess the effectiveness of metadata‐guided ComBat harmonization in improving cross‐center consistency. In this study, robustness was operationally defined as reproducibility quantified using intraclass correlation coefficients (ICC) across varying dose levels and acquisition‐related conditions. By systematically analyzing ICC‐based agreement across dose levels, anatomical organs, and harmonization scenarios, we sought to identify radiomic features that remain stable under count reduction and to quantify the extent to which harmonization enhances their stability. Robustness and reproducibility are necessary prerequisites for downstream quantitative modeling; however, they do not by themselves establish predictive performance or clinical utility, which require separate task‐specific validation.

## 2 MATERIALS AND METHODS

### 2.1 Study design

This retrospective multi‐center study was conducted to evaluate the dose‐dependent robustness of total‐body PET radiomic features and the impact of metadata‐guided harmonization strategies on feature reproducibility. PET datasets from two institutions were analyzed. For each patient, multiple retrospectively simulated dose levels were reconstructed from list‐mode acquisitions, followed by deep learning‐based organ segmentation and radiomics feature extraction. Feature harmonization was performed under different metadata settings, and reproducibility assessed using intraclass correlation analysis across predefined experimental scenarios. The overall study workflow is illustrated in Figure 1.

**FIGURE 1:**
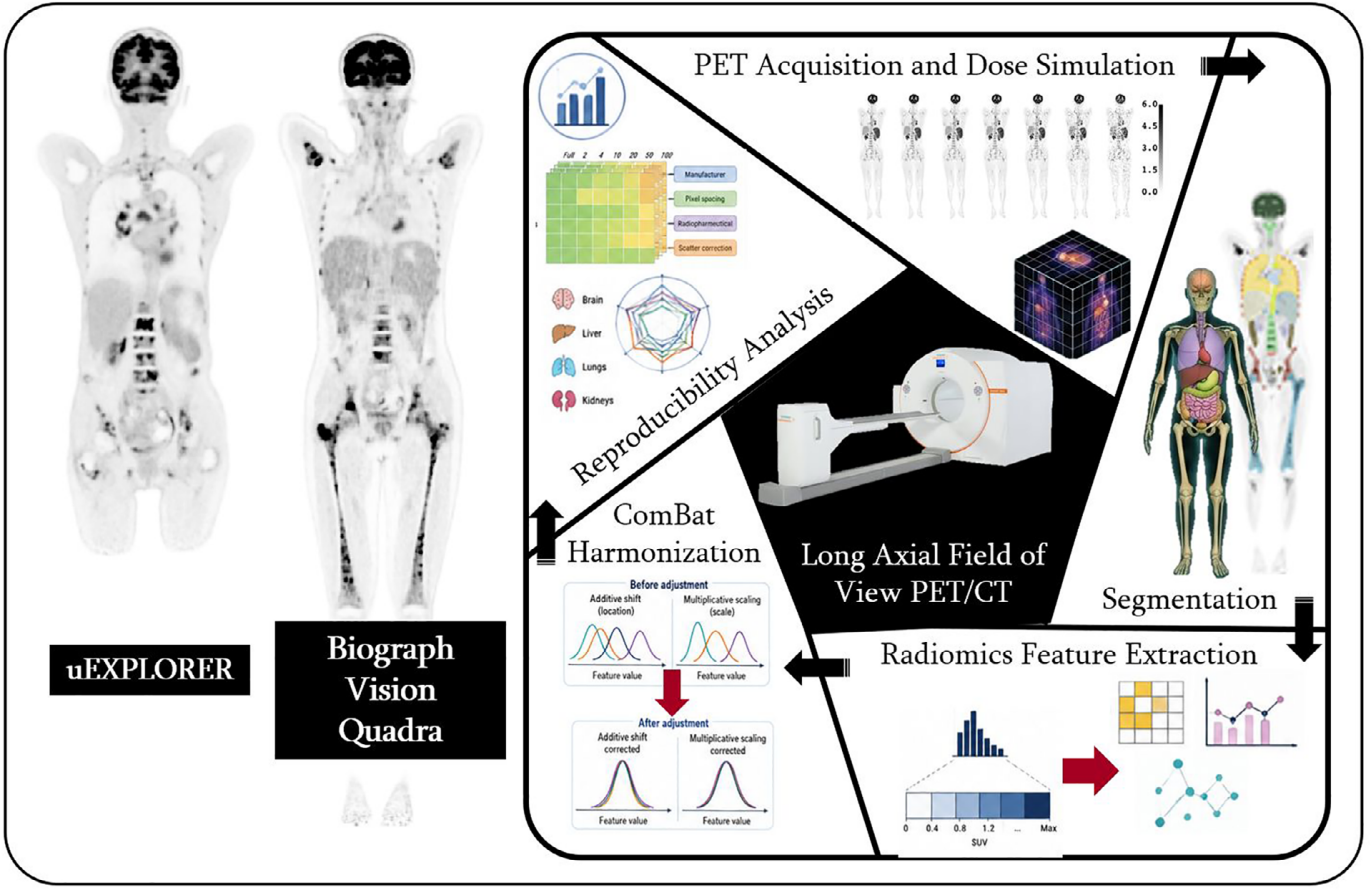
Overview of the study workflow. Total‐body PET data acquired at two centers using the uEXPLORER and Biograph Vision Quadra systems underwent retrospective reduced‐count simulation at multiple dose‐reduction factors. Whole‐organ masks were generated directly from the standard full‐count PET reconstruction using a pretrained PET‐based segmentation framework and were applied unchanged to all corresponding reduced‐count reconstructions. Radiomic features based on IBSI‐standardized definitions were subsequently extracted, followed by feature‐level ComBat harmonization using different acquisition‐related batch variables. Reproducibility across dose levels, anatomical regions, and harmonization strategies was assessed using the intraclass correlation coefficient.

### 2.2 PET acquisition and dose simulation

This study utilized a multi‐center total‐body PET dataset acquired under a collaborative data‐sharing agreement between the University of Bern (Switzerland) and Ruijin Hospital, Shanghai Jiao Tong University School of Medicine (China).^38^ The dataset comprised whole‐body PET examinations from 550 patients scanned on two digital total‐body PET/CT systems: the uEXPLORER scanner (United Imaging Healthcare, Shanghai, China) and the Siemens Biograph Vision Quadra (Siemens Healthineers, Erlangen, Germany). Examinations included multiple radiotracers, namely [^18^F]‐FDG, [^18^F]‐PSMA, [^68^Ga]‐DOTA‐TOC, and [^68^Ga]‐DOTA‐TATE. Center‐specific administered activities are summarized in Table 1.

**TABLE 1:** Key acquisition and reconstruction differences between centers.

Parameter	Center A (uEXPLORER)	Center B (Biograph Quadra)
Manufacturer	United Imaging	Siemens Healthineers
Voxel size (mm^3^)	1.66 × 1.66 × 2.88	1.65 × 1.65 × 1.65
Reconstruction	TOF + PSF	TOF + PSF
Scatter correction	Model‐based	Model‐based (relative scatter scaling)
Mean administered activity (MBq)	193 ± 38	215 ± 49

All PET data were acquired in list mode, enabling retrospective simulation of reduced‐count conditions. Reduced‐count reconstructions were generated by rebinning events from shorter contiguous temporal windows centered at the midpoint of the original acquisition, with the retained duration reduced according to the corresponding dose‐reduction factor. dose reduction factors (DRFs) of 2, 4, 10, 20, 50, and 100 represented approximately 50%, 25%, 10%, 5%, 2%, and 1% of the full‐count acquisition duration, respectively. Within each scan, the same reconstruction parameters and post‐processing procedures were applied to the full‐count and reduced‐count datasets. Centering maintained a common temporal midpoint across count levels and avoided preferential sampling of only the early or late portion of the acquisition. This temporal‐window subsampling procedure differs from random event thinning, in which a predefined fraction of events is retained probabilistically across the complete acquisition interval. Because all reduced‐count reconstructions were derived from the same original list‐mode acquisition, they remained intrinsically co‐registered with the corresponding full‐count reference image. Representative examples of the full‐count and simulated reduced‐count PET images across the evaluated DRFs are shown in Figure 2.

**FIGURE 2:**
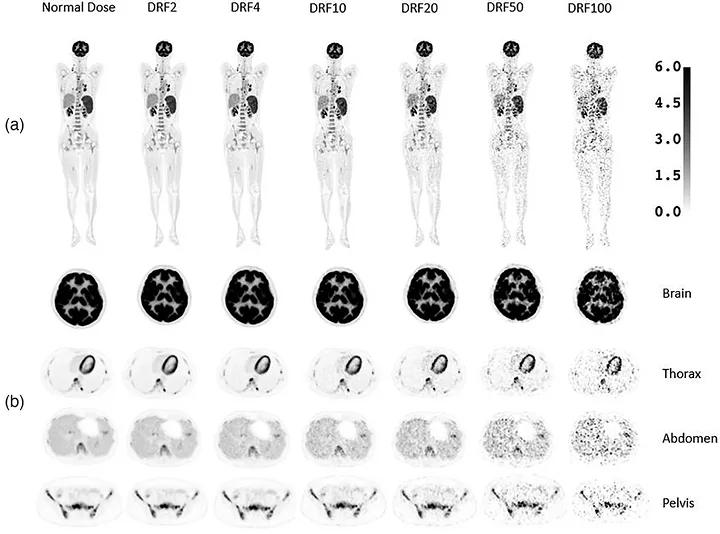
Representative PET images across retrospectively simulated dose‐reduction levels. Panel A shows total‐body maximum‐intensity‐projection images from one representative patient, displayed from left to right at the standard dose and at DRF 2, 4, 10, 20, 50, and 100. Increasing DRF corresponds to progressively reduced count statistics and increased image noise. Panel B shows representative axial views of the brain, thorax, abdomen, and pelvis from a second representative patient at the same dose‐reduction levels, illustrating local noise amplification and reduced anatomical conspicuity under severe count reduction. Within each panel, anatomical alignment was preserved because all reduced‐count images were reconstructed by retrospective temporal subsampling of the same list‐mode acquisition. Images within each panel were displayed using a consistent intensity window.

Acquisition and reconstruction parameters were extracted directly from the DICOM metadata of each scan. The two centers differed in manufacturer model, voxel size, slice thickness, reconstruction algorithm, and correction schemes (Table 1). Available demographic, anthropometric, radiopharmaceutical, and imaging acquisition characteristics of the two study cohorts are summarized in Table S1.

### 2.3 Image segmentation

Automatic multi‐organ segmentation was performed directly on the standard full‐count PET reconstruction using pretrained PET‐based models implemented within the nnU‐Net v2 framework and publicly available on GitHub.^39^ The deployed models used the three‐dimensional full‐resolution configuration and were applied using a predefined model‐assignment and inference workflow. No cohort‐specific retraining, fine‐tuning, parameter tuning, or modification of the segmentation workflow was performed. A total of 21 anatomical regions were segmented, including brain, eyes, clavicle, ribs, vertebrae, sacral bone, femur, hips, aorta, adrenal glands, colon, esophagus, stomach, pancreas, gallbladder, liver, lungs, kidneys, bladder, heart, and erector spinae muscles. All standard full‐count PET images were processed using the same segmentation software version and fixed preprocessing, inference, and post‐processing settings.

For each patient, segmentation was performed once on the standard full‐count PET reconstruction, and the resulting multi‐organ segmentation mask was retained unchanged and applied to all corresponding reduced‐count reconstructions. Because all reduced‐count images were reconstructed from the same original list‐mode acquisition, the dose levels remained intrinsically co‐registered. Fixing the mask across dose conditions was an intentional design choice aimed at isolating the effect of PET count reduction on intensity‐ and texture‐derived radiomic features. Consequently, dose‐dependent segmentation uncertainty was not included in the reproducibility analysis, and the resulting ICC estimates should be interpreted as conditional on identical segmentation masks across dose levels and as best‐case estimates of features reproducibility.

All segmentation masks were visually inspected for gross anatomical plausibility before radiomic feature extraction, without subsequent manual modification. Detailed model provenance, preprocessing, model‐assignment, inference, quality‐control, and mask‐application parameters are provided in Table S2. A schematic overview of the segmentation framework and representative multi‐organ segmentation outputs is presented in Figure 3.

**FIGURE 3:**
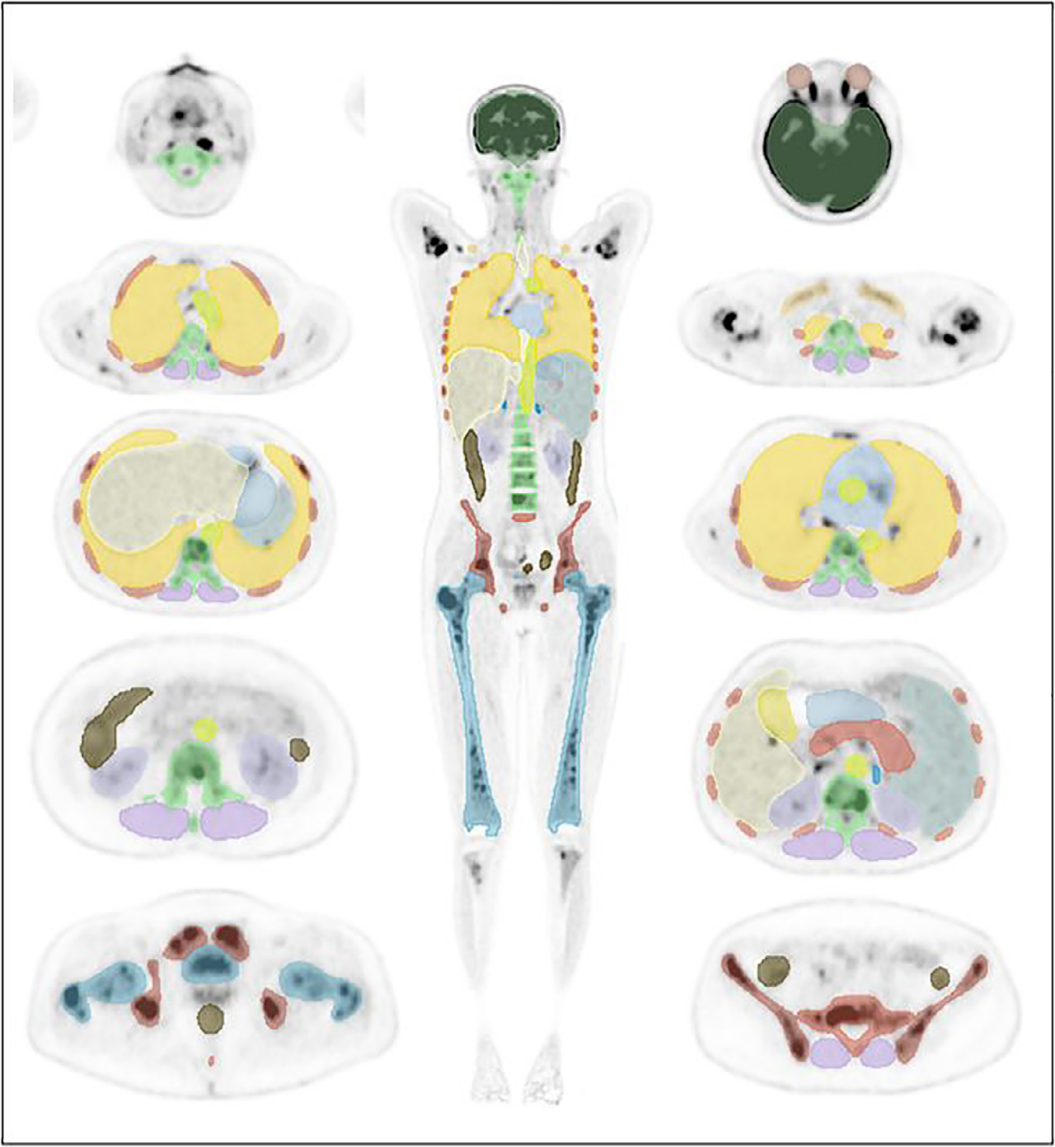
Representative examples of automated PET‐based multi‐organ segmentation generated from the standard full‐count PET reconstruction using the pretrained nnU‐Net framework. Color‐coded overlays illustrate segmented anatomical regions across axial slices and whole‐body projection views, including brain, thoracic organs, abdominal organs, skeletal structures, and pelvic regions.

### 2.4 Radiomics extraction

Radiomics feature extraction was performed using the open‐source PyRadiomics library^40^ (version 3.1.0) in Python, in accordance with the Image Biomarker Standardization Initiative (IBSI) guidelines.^41^, ^42^ PET images were analyzed at their native voxel spacing without isotropic resampling because voxel spacing was one of the acquisition‐related factors evaluated in the harmonization analysis. No intensity‐based re‐segmentation was applied, and the PET‐derived whole‐organ masks generated from the standard full‐count reconstruction were retained unchanged across all dose‐reduction levels within each patient. A total of 107 radiomic features were extracted from each segmented organ volume. The extracted features comprised 14 shape features, 18 first‐order statistics features, 24 Gray Level Co‐occurrence Matrix (GLCM) features, 16 Gray Level Run Length Matrix (GLRLM) features, 16 Gray Level Size Zone Matrix (GLSZM) features, 14 Gray Level Dependence Matrix (GLDM) features, and 5 Neighboring Gray Tone Difference Matrix (NGTDM) features. Prior to texture computation, voxel intensities were discretized using a fixed bin width of 0.4 SUV units.

### 2.5 Harmonization strategy

To mitigate acquisition‐related variability, radiomics feature harmonization was performed using a Python implementation of the standard ComBat model without covariates.^43^, ^44^ ComBat estimates additive and multiplicative batch effects at the feature level and adjusts feature distributions accordingly. Four ComBat configurations and one non‐harmonized baseline were evaluated:

1. Manufacturer (scanner type)
2. Pixel spacing
3. Radiopharmaceutical‐based exploratory sensitivity analysis
4. Scatter correction method
5. Non‐harmonized baseline (no ComBat adjustment)

Manufacturer, pixel spacing, and scatter‐correction method were treated as technical batch variables because they may introduce acquisition‐related variability in PET radiomics.^45^, ^46^, ^47^ Radiopharmaceutical identity was evaluated separately as an exploratory analysis because [^1^ ^8^F]FDG, [^1^ ^8^F]PSMA, [^6^ ^8^Ga]DOTA‐TOC, and [^6^ ^8^Ga]DOTA‐TATE exhibit distinct uptake mechanisms and biodistribution patterns. Therefore, tracer‐based ComBat adjustment may remove biologically meaningful variation and was interpreted descriptively rather than as primary evidence of technical harmonization effectiveness. For each configuration, ComBat was applied to the full radiomics feature matrix across all patients. The resulting feature sets were subsequently subjected to ICC‐based reproducibility analysis.

### 2.6 Reproducibility analysis

Feature reproducibility was quantified using the ICC based on a two‐way random‐effects model with absolute agreement (ICC(2,1)).^48^, ^49^ This model was selected because both subjects and measurement conditions were considered random effects, and absolute agreement was required to assess interchangeability of radiomics feature values across varying acquisition conditions. ICC was computed separately for each radiomics feature under multiple predefined experimental scenarios. In all analyses, subjects were defined as individual patients (*n*), while repeated measurements (*k*) corresponded to varying experimental conditions, depending on the specific comparison being performed.

To systematically investigate the effects of dose level, harmonization, and anatomical region, the analyses were organized into six predefined scenarios:

1. Non‐harmonized dose robustness: cross‐dose ICC was calculated within each anatomical region using the non‐harmonized data,
2. Harmonized dose robustness: cross‐dose ICC was calculated within each anatomical region after each ComBat adjustment,
3. Harmonization impact at fixed dose levels: ICC results were compared across harmonization settings within each anatomical region and dose level,
4. Organ‐dependent dose robustness without harmonization: cross‐dose ICC patterns were compared across anatomical regions using the non‐harmonized data,
5. Organ‐dependent dose robustness after harmonization: cross‐dose ICC patterns were compared across anatomical regions after each ComBat adjustment,
6. Combined dose–organ–harmonization assessment: reproducibility patterns were jointly summarized across dose levels, anatomical regions, and harmonization settings.

ICC values were calculated for all 107 radiomic features, and scenario‐specific summaries were generated across feature classes, anatomical regions, dose levels, and harmonization settings.

### 2.7 Statistical analysis

All analyses were performed in Python (v3.11) using open‐source scientific libraries. Data handling and numerical computations were conducted using pandas and numpy, and visualizations were generated using matplotlib and seaborn. The ICC(2,1) was implemented following the two‐way random‐effects ANOVA model described by Koo and Li.^49^ ICC values were calculated independently for each radiomics feature under each predefined experimental scenario. To identify dose‐reduction thresholds for individual radiomic features, an additional reference‐based pairwise analysis was performed. ICC(2,1) values were calculated between the standard‐dose reconstruction and each reduced‐count reconstruction at DRF 2, 4, 10, 20, 50, and 100. For each feature and anatomical region, the first‐failure DRF was defined as the lowest dose‐reduction factor at which ICC decreased below 0.75. Features maintaining ICC ≥ 0.75 at all evaluated dose levels were classified as showing no failure through DRF 100. Shape features were excluded from interpretation because identical PET‐derived masks were used across all dose conditions.

For visualization, mean ICC values were aggregated within feature classes and displayed using heatmaps across dose levels, harmonization settings, and anatomical regions. Feature‐level ICC values with 95% confidence intervals were reported for features demonstrating good or excellent reproducibility. Given the methodological focus of this study, analyses were descriptive and reproducibility‐oriented rather than inferential.

## 3 RESULTS

The results are presented according to the six predefined reproducibility scenarios described in Section 2.6. An overall feature‐level summary is followed by scenario‐specific analyses of dose robustness, harmonization effects, organ‐dependent variability, and their combined patterns.

### 3.1 Overall feature‐level reproducibility

Table 2 summarizes feature‐level reproducibility across anatomical regions. Overall, 35 of 107 radiomic features (32.7%) achieved good or excellent reproducibility (ICC ≥ 0.75) in at least one region. Reproducible features were predominantly drawn from first‐order and GLCM families. The Mean first‐order feature demonstrated the broadest stability, achieving ICC ≥ 0.75 across 10 anatomical regions. Among texture metrics, Sum Average and Joint Average (GLCM) showed consistent reproducibility in 8–9 regions. Reproducibility patterns varied substantially by anatomical site. The bladder exhibited the greatest concentration of stable features, encompassing metrics from all feature families. Skeletal structures, such as vertebrae and femur, also demonstrated cross‐family stability. In contrast, vascular regions (e.g., aorta, kidneys) showed limited feature‐level reproducibility, with only a small subset of features exceeding the ICC threshold.

**TABLE 2:** Radiomic features demonstrating good or excellent reproducibility (ICC ≥ 0.75) across dose levels and harmonization conditions.

Feature Name	Feature Group	Anatomical Region
**Dependence Entropy**	GLDM	Vertebrae
**Maximum Probability**	GLCM	Vertebrae—Femur
**Mean**	First‐order	Vertebrae—Sacral Bone—Colon—Bladder—Pancreas—Aorta—Femur—Erector Spinae—hips—Kidneys
**Large Dependence Low Gray Level Emphasis**	GLDM	Vertebrae—Aorta—Erector Spinae—hips
**Sum Average**	GLCM	Vertebrae—Sacral Bone—Colon—Bladder—Femur—Erector Spinae—hips– Kidneys—Pancreas
**Autocorrelation**	GLCM	Vertebrae—Sacral Bone—Bladder—Pancreas—Femur—
**Strength**	NGTDM	Vertebrae—Bladder—Pancreas
**Joint Average**	GLCM	Vertebrae—Sacral Bone—Colon—Bladder—Pancreas—Femur—Erector Spinae—hips—Kidneys
**Sum Entropy**	GLCM	Colon—
**90 Percentile**	First‐order	Colon—Bladder—Femur
**Gray Level Non‐Uniformity**	GLDM	Colon—Stomach—Femur
**Contrast**	NGTDM	Bladder
**Small Area High Gray Level Emphasis**	GLSZM	Bladder—Pancreas
**Gray Level Variance**	GLDM	Bladder
**Difference Variance**	GLCM	Bladder
**Gray Level Variance**	GLSZM	Bladder—Pancreas
**High Gray Level Run Emphasis**	GLRLM	Bladder—Pancreas
**Sum Squares**	GLCM	Bladder
**Variance**	First‐order	Bladder
**Root Mean Squared**	First‐order	Bladder—Pancreas
**Gray Level Variance**	GLRLM	Bladder
**Total Energy**	First‐order	Bladder—Pancreas—Femur
**Complexity**	NGTDM	Bladder
**Long Run High Gray Level Emphasis**	GLRLM	Bladder
**High Gray Level Emphasis**	GLDM	Bladder—Pancreas
**High Gray Level Zone Emphasis**	GLSZM	Bladder—Pancreas
**Difference Average**	GLCM	Bladder
**Energy**	First‐order	Bladder—Pancreas—Femur
**Short Run High Gray Level Emphasis**	GLRLM	Bladder
**Cluster Shade**	GLCM	Bladder
**Small Dependence High Gray Level Emphasis**	GLDM	Bladder
**Cluster Prominence**	GLCM	Bladder
**Joint Energy**	GLCM	Femur
**Gray Level Non‐Uniformity**	GLRLM	Femur
**Interquartile Range**	First‐order	Femur

*Note*: The table lists feature name, corresponding radiomics family, and anatomical regions in which each feature achieved high stability. Reported regions indicate where features maintained strong cross‐dose agreement, highlighting organ‐dependent robustness patterns across first‐order and texture feature classes.

Table 3 provides the corresponding ICC values and 95% confidence intervals for features demonstrating good or excellent reproducibility. ICC values ranged from 0.751 to 0.906. The highest value was observed for GLCM Cluster Prominence in the bladder (ICC = 0.906, 95% CI: 0.857–0.948). Several additional bladder‐derived texture features exceeded an ICC of 0.88. First‐order Mean achieved ICC values of 0.845 in the colon, 0.893 in the erector spinae, and 0.820 in the femur. Sum Average and Joint Average also exceeded 0.80 in several skeletal and abdominal regions. Overall, high‐contrast and skeletal structures generally showed stronger agreement than vascular and lower‐contrast soft‐tissue regions. In addition to the 35 features achieving good or excellent reproducibility, the remaining features were evaluated for poor or variable performance. These features were not uniformly poorly reproducible, because some demonstrated moderate or organ‐dependent agreement. Features most frequently showing ICC values below 0.50 were predominantly derived from the GLSZM, GLRLM, and GLDM families, including Zone Percentage, Zone Variance, Large Area High Gray Level Emphasis, Large Area Low Gray Level Emphasis, Small Area Low Gray Level Emphasis, Small Dependence Low Gray Level Emphasis, Size Zone Non‐Uniformity Normalized, and Run Length Non‐Uniformity Normalized. The first‐order 10th Percentile feature also showed poor reproducibility in several anatomical regions.

**TABLE 3:** Radiomic features demonstrating good and excellent reproducibility (ICC ≥ 0.75) across anatomical regions, including corresponding ICC(2,1) values and 95% confidence intervals.

Anatomical Region	Feature Name	Feature Group	ICC	95% CI Lower	95% CI Upper
**Vertebrae**	Dependence Entropy	GLDM	0.754	0.664	0.860
	Maximum Probability	GLCM	0.753	0.658	0.856
	Mean	First‐order	0.826	0.742	0.899
	Large Dependence Low Gray Level Emphasis	GLDM	0.782	0.724	0.890
	Sum Average	GLCM	0.826	0.742	0.899
	Autocorrelation	GLCM	0.771	0.682	0.869
	Strength	NGTDM	0.776	0.724	0.890
	Joint Average	GLCM	0.823	0.738	0.897
**Sacral Bone**	Mean	First‐order	0.779	0.684	0.870
	Sum Average	GLCM	0.779	0.685	0.871
	Autocorrelation	GLCM	0.803	0.750	0.903
	Joint Average	GLCM	0.777	0.681	0.869
**Colon**	Mean	First‐order	0.845	0.765	0.909
	Sum Entropy	GLCM	0.777	0.698	0.878
	90 Percentile	First‐order	0.788	0.750	0.903
	Sum Average	GLCM	0.844	0.764	0.909
	Joint Average	GLCM	0.840	0.758	0.906
	Gray Level Non‐Uniformity	GLDM	0.793	0.699	0.878
**Bladder**	Contrast	NGTDM	0.899	0.848	0.945
	Small Area High Gray Level Emphasis	GLSZM	0.888	0.829	0.937
	Gray Level Variance	GLDM	0.882	0.822	0.934
	Mean	First‐order	0.762	0.687	0.872
	Difference Variance	GLCM	0.894	0.840	0.942
	Gray Level Variance	GLSZM	0.897	0.841	0.942
	High Gray Level Run Emphasis	GLRLM	0.871	0.807	0.928
	90 Percentile	First‐order	0.814	0.743	0.899
	Sum Average	GLCM	0.772	0.695	0.876
	Sum Squares	GLCM	0.886	0.826	0.936
	Variance	First‐order	0.884	0.824	0.935
	Root Mean Squared	First‐order	0.803	0.732	0.894
	Gray Level Variance	GLRLM	0.883	0.822	0.934
	Total Energy	First‐order	0.866	0.802	0.926
	Autocorrelation	GLCM	0.873	0.810	0.929
	Complexity	NGTDM	0.865	0.800	0.925
	Strength	NGTDM	0.870	0.806	0.928
	Long Run High Gray Level Emphasis	GLRLM	0.868	0.804	0.927
	High Gray Level Emphasis	GLDM	0.874	0.811	0.930
	Joint Average	GLCM	0.772	0.695	0.876
	High Gray Level Zone Emphasis	GLSZM	0.884	0.823	0.935
	Difference Average	GLCM	0.842	0.776	0.914
	Energy	First‐order	0.796	0.721	0.889
	Short Run High Gray Level Emphasis	GLRLM	0.871	0.808	0.928
	Cluster Shade	GLCM	0.866	0.801	0.925
	Small Dependence High Gray Level Emphasis	GLDM	0.884	0.824	0.935
	Cluster Prominence	GLCM	0.906	0.857	0.948
**Pancreas**	Small Area High Gray Level Emphasis	GLSZM	0.802	0.721	0.889
	Mean	First‐order	0.806	0.710	0.883
	Gray Level Variance	GLSZM	0.812	0.740	0.898
	High Gray Level Run Emphasis	GLRLM	0.751	0.697	0.877
	Sum Average	GLCM	0.765	0.668	0.862
	Root Mean Squared	First‐order	0.777	0.706	0.882
	Total Energy	First‐order	0.861	0.811	0.930
	Autocorrelation	GLCM	0.822	0.761	0.908
	Strength	NGTDM	0.841	0.774	0.913
	High Gray Level Emphasis	GLDM	0.773	0.715	0.886
	Joint Average	GLCM	0.765	0.668	0.862
	High Gray Level Zone Emphasis	GLSZM	0.803	0.720	0.888
	Energy	First‐order	0.844	0.789	0.920
**Aorta**	Mean	First‐order	0.752	0.653	0.854
	Large Dependence Low Gray Level Emphasis	GLDM	0.784	0.695	0.876
**Stomach**	Gray Level Non‐Uniformity	GLDM	0.816	0.727	0.891
**Femur**	Maximum Probability	GLCM	0.771	0.680	0.868
	Joint Energy	GLCM	0.770	0.690	0.873
	Mean	First‐order	0.820	0.731	0.894
	90 Percentile	First‐order	0.808	0.756	0.905
	Sum Average	GLCM	0.805	0.726	0.891
	Total Energy	First‐order	0.807	0.797	0.924
	Autocorrelation	GLCM	0.820	0.792	0.921
	Joint Average	GLCM	0.799	0.715	0.886
	Energy	First‐order	0.772	0.759	0.907
	Gray Level Non‐Uniformity	GLRLM	0.771	0.704	0.880
	Interquartile Range	First‐order	0.800	0.734	0.895
	Gray Level Non‐Uniformity	GLDM	0.854	0.782	0.917
**Erector Spinae**	Mean	First‐order	0.893	0.838	0.941
	Large Dependence Low Gray Level Emphasis	GLDM	0.785	0.736	0.896
	Sum Average	GLCM	0.891	0.829	0.937
	Joint Average	GLCM	0.878	0.811	0.930
**hips**	Mean	First‐order	0.843	0.780	0.916
	Large Dependence Low Gray Level Emphasis	GLDM	0.756	0.699	0.878
	Sum Average	GLCM	0.834	0.772	0.912
	Joint Average	GLCM	0.831	0.766	0.910
**Kidneys**	Mean	First‐order	0.787	0.691	0.874
	Sum Average	GLCM	0.752	0.647	0.850
	Joint Average	GLCM	0.752	0.648	0.851

*Note*: The table reports feature name, radiomics family, and organ‐specific stability, highlighting region‐dependent robustness patterns. Bladder and skeletal structures exhibited the highest ICC values, whereas vascular and soft‐tissue organs showed comparatively lower reproducibility ranges.

### 3.2 Cross‐dose and organ‐dependent robustness

Figure 4 summarizes feature‐family‐level cross‐dose reproducibility for each anatomical region under the non‐harmonized baseline and the four harmonization configurations. Under non‐harmonized conditions, median cross‐dose ICC values across the evaluable anatomical regions were 0.401 for first‐order features, 0.404 for GLCM, 0.310 for GLDM, 0.333 for GLRLM, 0.347 for GLSZM, and 0.368 for NGTDM. Hence, all non‐shape feature families demonstrated poor median cross‐dose reproducibility at the family level. First‐order features achieved ICC ≥ 0.75 in 3 of 19 evaluable regions under the non‐harmonized condition.

**FIGURE 4:**
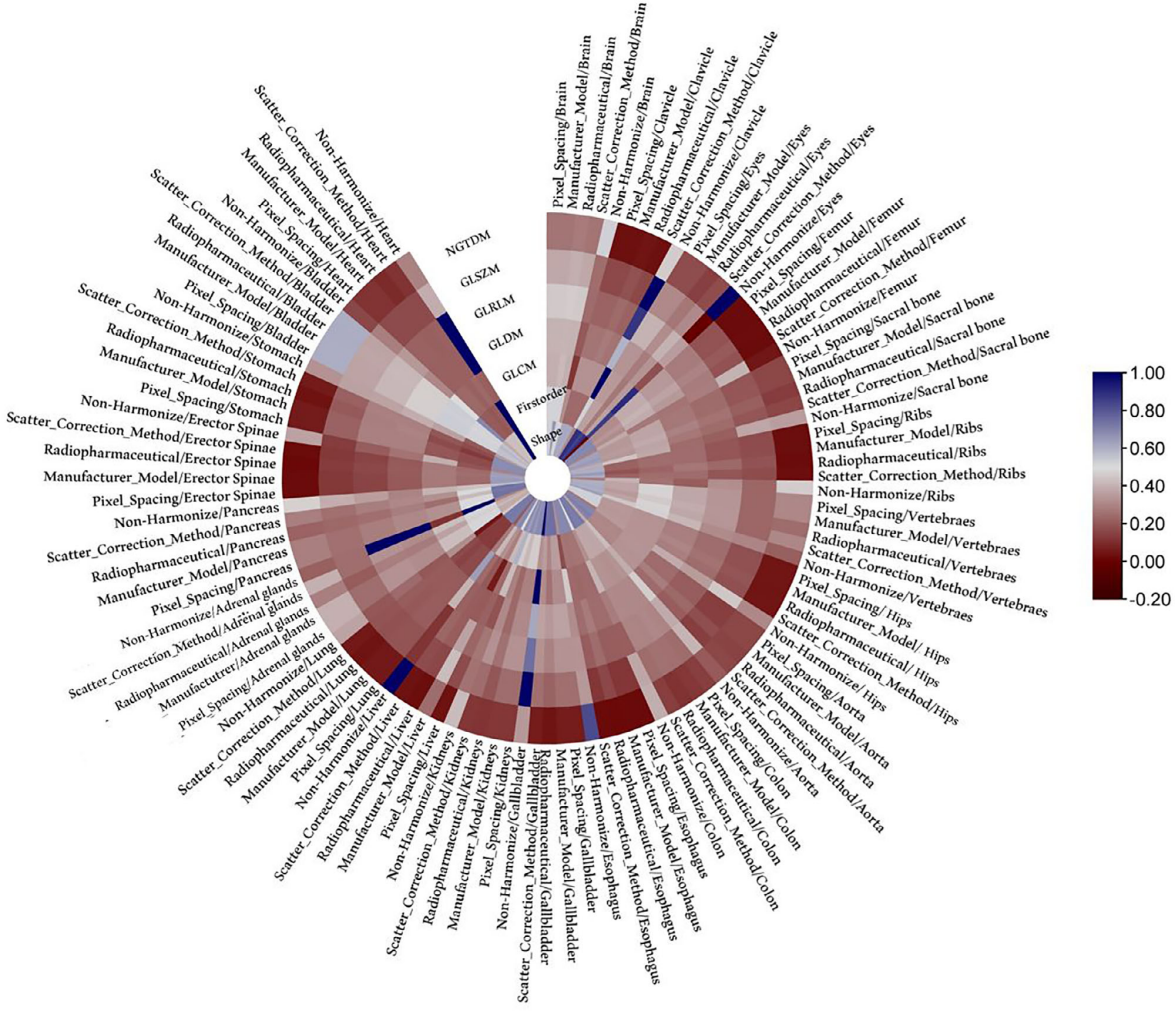
Circular heatmap of cross‐dose intraclass correlation coefficients across harmonization settings and anatomical regions. Each angular segment represents a specific combination of anatomical region and processing condition, including the non‐harmonized baseline, pixel‐spacing–based, manufacturer‐based, radiopharmaceutical‐based, and scatter correction–based adjustments. For each organ–harmonization combination, ICC(2,1) was calculated across dose‐reduction levels. Radial bands represent radiomics feature families, including shape, first‐order, GLCM, GLDM, GLRLM, GLSZM, and NGTDM.

Cross‐dose reproducibility remained dependent on both anatomical region and adjustment strategy after harmonization. Across all evaluable non‐shape organ–feature‐family combinations, the overall median ICC was 0.364 for the non‐harmonized baseline, compared with 0.265 for manufacturer‐based adjustment, 0.258 for pixel‐spacing–based adjustment, 0.258 for scatter correction–based adjustment, and 0.253 for radiopharmaceutical‐based adjustment. The corresponding median first‐order ICC values were 0.346, 0.347, 0.347, and 0.330, respectively. No region achieved a first‐order family‐level ICC ≥ 0.75 after manufacturer, pixel‐spacing, scatter correction, or radiopharmaceutical‐based adjustment. Therefore, no harmonization configuration uniformly improved cross‐dose reproducibility across anatomical regions and feature families. Among the technical adjustments, manufacturer, pixel‐spacing, and scatter correction‐based configurations produced broadly similar overall reproducibility patterns.

The exploratory radiopharmaceutical‐based adjustment showed organ‐dependent changes in ICC; however, these effects were not interpreted as evidence of improved technical harmonization because tracer identity contains biologically meaningful variation. Shape features were not considered informative for assessing sensitivity to PET count reduction because their values were determined by identical PET‐derived masks across dose levels.

The omnibus cross‐dose ICC analysis presented in Figure 4 and the reference‐based first‐failure analysis addressed complementary but distinct aspects of reproducibility. Figure 4 summarizes agreement across all evaluated dose levels simultaneously, whereas the first‐failure analysis compares each reduced‐count reconstruction separately with the standard‐dose reference and identifies the earliest DRF at which ICC falls below 0.75. Consequently, the results of these two analyses are not directly interchangeable: a feature family may show a low omnibus ICC across the complete dose range while retaining acceptable pairwise agreement with the standard‐dose image at several individual DRFs.

The first‐failure analysis showed organ‐ and feature‐dependent variation. At the feature‐family level, first‐order and GLCM features showed no failure through DRF 100 in 15 of 21 and 18 of 21 regions, respectively. Among the selected features, Total Energy and Autocorrelation remained reproducible through DRF 100 in all regions, whereas Mean, Sum Average, and Joint Average first failed at DRF 4 in the kidneys and liver. Energy showed earlier failures at DRF 2 in the aorta, esophagus, and lungs. Detailed results are provided in Tables S3 and S4.

### 3.3 Harmonization agreement at fixed dose levels

Figure 5 presents feature‐family‐level agreement across harmonization settings for each anatomical region and dose level. Excluding shape features, the median inter‐method ICC across all evaluable organ–feature‐family combinations was 0.934 at the standard dose, 0.908 at DRF 20, 0.869 at DRF 50, and 0.906 at DRF 100. ICC values of at least 0.75 were observed in 126 of 126 evaluable combinations at the standard dose, 101 of 101 at DRF 20, 101 of 101 at DRF 50, and 118 of 122 at DRF 100.

**FIGURE 5:**
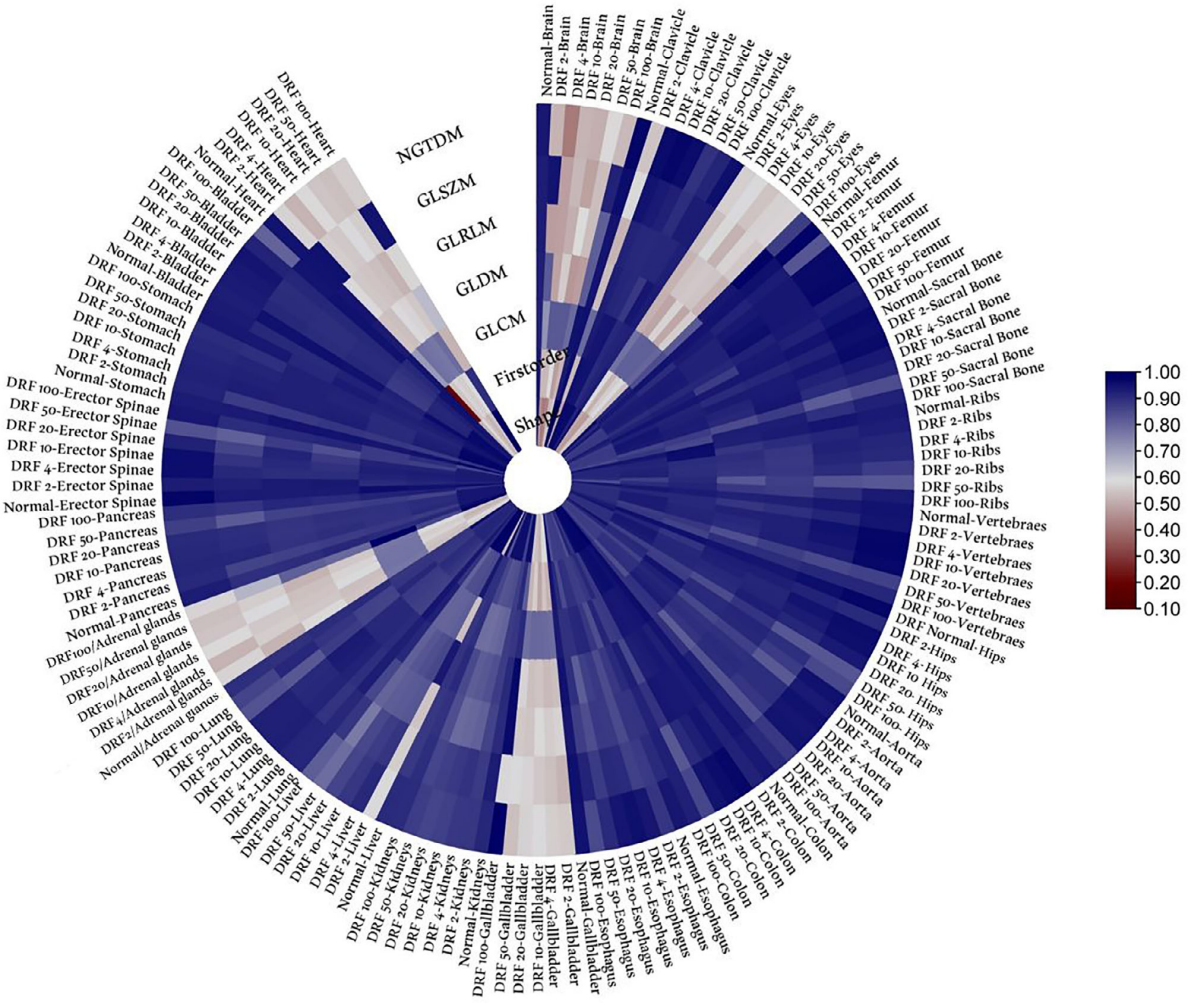
Circular heatmap of cross‐harmonization intraclass correlation coefficients across dose‐reduction levels and anatomical regions. Each angular segment represents a specific combination of anatomical region and dose level, from the standard‐dose reconstruction to DRF 100. For each organ–dose combination, ICC(2,1) was calculated across the evaluated harmonization settings. Radial bands represent radiomics feature families, including shape, first‐order, GLCM, GLDM, GLRLM, GLSZM, and NGTDM.

Inter‐method agreement therefore exhibited a non‐monotonic and heterogeneous pattern. The overall median ICC reached its lowest value at DRF 50, all evaluable organ–feature‐family combinations remained within the good or excellent reproducibility range. At DRF 100, the median ICC increased again, although four individual combinations fell below 0.75. These comprised GLSZM in the gallbladder and adrenal glands, and GLDM and GLRLM in the heart.

First‐order features maintained good‐to‐excellent median inter‐method agreement across all dose levels, with median ICC values ranging from 0.870 at DRF 50 to 0.938 at the full dose. The median ICC subsequently increased to 0.902 at DRF 100, indicating a non‐monotonic pattern. Shape‐feature results were not interpreted as evidence of dose robustness because identical PET‐derived masks were used across all dose conditions. The magnitude of inter‐method agreement also varied among anatomical regions. At DRF 100, organ‐level median ICC values ranged from 0.772 in the heart and 0.808 in the adrenal glands to 0.935 in the gallbladder and 0.947 in the bladder.

Joint interpretation of Figures 4 and 5 demonstrated that radiomic reproducibility was determined by the combined effects of dose reduction, anatomical region, feature family, and harmonization strategy. Moderate count reduction preserved selected first‐order and intensity‐associated features in several regions, whereas extreme count reduction predominantly affected higher‐order texture features. Harmonization reduced some acquisition‐related variability but did not uniformly compensate for noise‐induced instability or eliminate organ‐dependent differences.

## 4 DISCUSSION

This study systematically evaluated the robustness of quantitative PET radiomic features across multiple retrospectively simulated dose‐reduction levels and harmonization strategies in a multi‐center total‐body PET cohort. Several key findings emerged. First, dose reduction had a measurable and organ‐dependent impact on feature stability. While moderate dose reductions (DRF = 2–20) preserved reproducibility in many anatomical regions, extreme dose reductions (DRF = 50 and 100) resulted in substantial declines in texture feature robustness, particularly in soft tissue and vascular structures. In contrast, skeletal and high‐contrast organs demonstrated comparatively preserved stability. Second, a subset of radiomic features remained consistently robust across dose levels and harmonization conditions. Notably, first ‐order features, such as Mean, Energy and Total Energy, showed stable behavior across multiple anatomical regions. Several GLCM‐derived features (e.g., Sum Average, Joint Average) also maintained good reproducibility in selected organs. Overall, approximately one‐third of the extracted features demonstrated good or excellent reproducibility in at least one anatomical region. Third, harmonization substantially influenced reproducibility patterns. Harmonization influenced cross‐dose reproducibility in an organ‐specific manner but did not consistently improve feature stability across anatomical regions or compensate for extreme count reduction. These findings indicate that dose robustness and inter‐center variability interact in a non‐trivial and anatomically dependent manner.

The results have methodological implications for quantitative PET imaging and multi‐center radiomics research. The preservation of reproducibility in selected features under moderate dose reduction supports their further evaluation in task‐specific studies but does not establish their clinical usefulness. In total‐body PET, reduced injected activity or shorter acquisition times may potentially support longitudinal or repeated imaging applications; however, the clinical suitability of the identified features requires prospective and endpoint‐specific validation. Organ‐dependent degradation of higher‐order texture features under extreme dose reduction highlights the need for caution when applying radiomics in ultra‐low‐count scenarios. Feature selection strategies should prioritize dose‐robust metrics, especially in multi‐center studies. The observed variability across harmonization strategies further underscores the importance of standardized preprocessing and harmonization pipelines prior to pooling multi‐center data. Without such adjustments, non‐biological variability may confound downstream quantitative analyses. To facilitate practical implementation of these findings in clinical and research workflows, a structured synthesis of radiomics robustness across dose levels, anatomical regions, and harmonization strategies is provided in Table 4.

**TABLE 4:** Practical summary of radiomics robustness across dose reduction and harmonization conditions.

Domain	More Favorable Patterns	Context‐Dependent Patterns	Higher Sensitivity or Caution
**Feature Families**	Selected first‐order and intensity‐associated features, particularly Mean, Total Energy, and Energy; Autocorrelation also showed broad stability	Selected GLCM features, including Sum Average and Joint Average, whose stability may partly reflect collinearity with global intensity	Higher‐order features from the GLDM, GLRLM, GLSZM, and NGTDM families, particularly under severe count reduction
**Anatomical Regions**	Bladder, vertebrae, femur, and erector spinae generally contained the greatest number of reproducible features	Colon, pancreas, kidneys, sacral bone, and hips showed feature‐dependent reproducibility	Aorta, adrenal glands, esophagus, lungs, heart, and selected liver features showed greater susceptibility to count reduction
**Dose Levels (DRF)**	Selected intensity‐associated features remained reproducible under moderate dose reduction, and some remained stable through DRF 100	Reproducibility between DRF 20 and DRF 50 was strongly dependent on organ and feature type	DRF 50–100 increased the risk of instability, particularly for higher‐order texture features; changes were not uniformly monotonic
**Harmonization Strategies**	The non‐harmonized baseline showed the highest overall median cross‐dose ICC among the evaluated configurations	Manufacturer, pixel‐spacing, and scatter correction–based adjustments produced broadly similar but organ‐ and feature‐dependent patterns; agreement across harmonization settings remained generally high at fixed dose levels	No ComBat configuration uniformly improved cross‐dose reproducibility. Radiopharmaceutical‐based adjustment was exploratory because it may remove biologically meaningful tracer‐related variation
**Implications for Low‐Dose Quantitative PET**	Selected intensity‐associated features may be prioritized for further evaluation in low‐count and multi‐center studies	Feature selection should be organ‐specific, dose‐aware, and supported by task‐specific validation	Higher‐order texture features require caution in ultra‐low‐count imaging, and reproducibility should not be interpreted as evidence of predictive or clinical usefulness

Previous PET radiomics studies have reported sensitivity of texture features to noise and acquisition variability, particularly under reduced count statistics.^24^, ^26^, ^36^ Our findings are consistent with prior work demonstrating that first‐order metrics tend to exhibit greater robustness compared to higher‐order texture features in low‐dose conditions. Similarly, prior investigations of ComBat harmonization in PET imaging have shown reductions in inter‐scanner variability and improved feature consistency.^14^, ^28^, ^33^ The present study extends these findings by evaluating harmonization performance under simultaneous dose reduction and multi‐center variability in total‐body PET systems. Unlike earlier studies that examined either dose effects or harmonization independently, our analysis demonstrated their combined and organ‐specific interaction. These observations are consistent with our recent multi‐organ stability framework, highlighting the importance of organ‐specific evaluation for identifying robust radiomic biomarkers in low‐dose total‐body PET.^50^

The observed sensitivity of texture features to dose reduction can be attributed to their reliance on spatial gray‐level relationships. Reduced count statistics increase image noise and alter gray‐level distributions, disproportionately affecting second‐ and higher‐order statistical matrices (GLCM, GLDM, GLRLM, GLSZM, NGTDM). In contrast, first‐order features, which summarize global intensity distributions, appear less sensitive to local noise fluctuations. The high reproducibility of Sum Average and Joint Average should nevertheless be interpreted cautiously. These GLCM features may be partly collinear with first‐order Mean and may therefore act as surrogates of global intensity rather than independent measures of spatial heterogeneity. Because feature redundancy was not formally evaluated in the present study, the extent of this association cannot be determined. Future studies should assess feature‐level correlations and the incremental information provided by these texture metrics beyond conventional intensity measures. The relative robustness of skeletal and high‐contrast organs likely reflects greater organ‐to‐background contrast and more homogeneous uptake patterns, which reduce susceptibility to stochastic noise under count reduction. In particular, the high number of robust texture features observed in the bladder may be partly explained by urinary radiotracer accumulation, resulting in high local count density and strong contrast. Therefore, this finding may reflect favorable counting statistics rather than an intrinsic robustness of bladder texture features and should be interpreted cautiously across different tracers and imaging protocols. Importantly, harmonization altered feature distributions and reproducibility patterns in an organ‐ and feature‐dependent manner but did not uniformly improve cross‐dose agreement or fully compensate for stochastic noise introduced by extreme dose reduction. These findings suggest that acquisition‐related batch effects and noise‐induced instability may represent partially independent sources of variability. Radiopharmaceutical‐based ComBat requires cautious interpretation because tracer identity reflects biologically meaningful differences in uptake and biodistribution rather than purely technical variability. Treating radiopharmaceuticals as a batch factor may therefore remove genuine tracer‐dependent signal; accordingly, this analysis was considered exploratory, and future studies should use tracer identity as a biological covariate or perform tracer‐stratified harmonization.

Several limitations should be acknowledged. First, this retrospective study used centered temporal‐window subsampling of list‐mode data rather than prospectively acquired low‐injected‐dose scans. Although this approach preserves spatial co‐registration and count‐dependent noise characteristics, it primarily simulates shortened acquisition durations and is not fully equivalent to random event thinning or prospectively reduced injected activity. Consequently, the results may differ in the presence of temporal changes in tracer distribution, patient motion, or count rate. Second, the study included data from two centers and two total‐body PET systems. While this design enabled multi‐center evaluation, broader validation across additional scanners and institutions would strengthen generalizability. Although retaining native voxel spacing enabled direct evaluation of voxel‐spacing–related variability and its harmonization, the absence of resampling to a common isotropic voxel spacing may have increased texture‐feature variability and may limit comparability with spatially standardized radiomics pipelines. No intensity‐based re‐segmentation was applied; although alternative thresholds may yield different feature values, they could also introduce dose‐, organ‐, and tracer‐dependent changes in the effective analysis mask. Third, a single PET‐derived segmentation mask generated from the standard full‐count reconstruction was applied unchanged to all reduced‐count reconstructions for each patient. Despite the use of the same segmentation framework and predefined inference workflow across the study cohorts, residual between‐patient and between‐cohort variability related to PET image quality, tracer‐dependent biodistribution, scanner characteristics, organ contrast, anatomical differences, and segmentation errors cannot be completely excluded. Nevertheless, because the same mask was retained across all dose levels within each patient, segmentation variability did not contribute to within‐patient differences across dose‐reduction conditions. While this design isolated count‐related effects, it excluded dose‐dependent segmentation uncertainty; therefore, the reported ICC values represent conditional best‐case estimates, and the apparent stability of shape features primarily reflects the fixed mask geometry rather than intrinsic robustness to PET count‐related noise. Finally, reproducibility was evaluated using ICC alone. Nevertheless, ICC is widely accepted for robustness assessment, complementary metrics, such as coefficient of variation or test–retest validation could provide additional insights.

Reproducibility should not be equated with clinical usefulness. A radiomic feature may remain stable across dose levels and acquisition conditions while providing limited information for diagnosis, prognosis, treatment response, or predictive modeling. The present study did not evaluate associations with clinical outcomes or downstream model performance. Therefore, potential applications in theranostic decision support or digital twin frameworks should be regarded as future directions requiring task‐specific predictive validation, external evaluation, and demonstration of incremental clinical value. In particular, recent developments in instrumentation digital twins have highlighted the need for robust and reproducible quantitative imaging biomarkers to support reliable physics‐informed virtual representations of imaging systems and quantitative imaging workflows.^51^

Future work should include phantom‐based validation to disentangle scanner‐specific effects from biological variability. Prospective test–retest studies would further clarify true biological reproducibility under low‐dose conditions. Multi‐tracer and multi‐center validation studies are also needed to determine whether dose robustness patterns generalize across different radiopharmaceuticals and disease contexts. Development of standardized radiomics pipelines, including harmonization and dose‐aware feature selection, will be essential for reliable quantitative deployment in clinical and research environments. Integration of advanced noise‐aware deep learning reconstruction frameworks with radiomics robustness analysis may represent a promising direction for stabilizing quantitative biomarkers under ultra‐low‐count conditions.^52^

## 5 CONCLUSION

This study systematically evaluated the robustness of quantitative PET radiomic features across multiple retrospectively simulated dose‐reduction levels and harmonization strategies in a multi‐center total‐body PET setting. Our findings demonstrate that radiomics stability is strongly dependent on dose level, anatomical region, and feature class. While moderate dose reduction preserved reproducibility in a substantial subset of features, particularly selected first‐order and intensity‐associated features, extreme count reduction resulted in pronounced degradation of higher‐order texture features. Organ‐specific variability was evident, with skeletal and high‐contrast structures exhibiting greater robustness than vascular and soft tissue regions. ComBat‐based harmonization produced organ‐ and feature‐dependent changes in reproducibility patterns. Despite the generally high agreement among the outputs of different harmonization settings at fixed dose levels, no configuration consistently improved cross‐dose reproducibility relative to the non‐harmonized baseline, and harmonization did not fully compensate for instability introduced by severe dose reduction. Overall, these results highlight the importance of dose‐aware feature selection and metadata‐guided harmonization in multi‐center PET radiomics. Identification of dose‐robust and harmonization‐stable features provides a methodological basis for future quantitative imaging studies; however, their predictive performance and clinical utility require separate task‐specific validation.

## CONFLICT OF INTEREST STATEMENT

The authors declare that they have no conflicts of interest.

## Supporting information



Supporting Information

## Data Availability

The imaging data used in this study are not publicly available owing to ethical and institutional restrictions. The code developed for the analysis is available upon request from the corresponding author.
