# Supplementary material for: Impact of dose reduction and multi‐parametric harmonization on multi‐organ radiomics robustness in total‐body PET imaging: Methodological implications for quantitative theranostics
Source: Med Phys. 2026 Oct 22;53(11):e70731. doi: 10.1002/mp.70731 (PMC13691394; doi:10.1002/mp.70731)
Supplement: Supplementary file 1 [file MP-53-0-s001.pdf]

Supplementary Table S1. Clinical, demographic, and imaging characteristics of the two study cohorts.

Characteristic	Bern Center (Inselspital)	China Center (Ruijin Hospital)
Number of Patients	275	275
Sex		
Male	(45.3%)	(51.8%)
Female	(54.7%)	(48.2%)
Age (years)	61.8 ± 13.4 (range: 22–87)	58.1 ± 14.3 (range: 18–85)
Height (m)	1.67 ± 0.09	1.66 ± 0.09
Weight (kg)	66.2 ± 17.0	65.4 ± 13.6
BMI (kg/m²)	23.7 ± 5.0	23.7 ± 4.1
Radiopharmaceutical	[18F]-FDG, [18F]-PSMA, [68Ga]-DOTA-TOC, and [68Ga]-DOTA-TATE	[18F]-FDG, [18F]-PSMA, [68Ga]-DOTA-TOC, and [68Ga]-DOTA-TATE
Injected Dose (MBq)	215 ± 49 (range: 154–345)	193 ± 38 (range: 101–343)
Scanner	Siemens Biograph Vision Quadra	UIH uEXPLORER
Reconstruction Method	PSF+TOF 4i5s	3D iterative TOF + PSF
Scatter Correction	Model-based, relative scatter scaling	Model-based
Attenuation Correction	CT-based	CT-based
Voxel size (mm³)	1.65 × 1.65 × 1.65	1.66 × 1.66 × 2.88
Acquisition (per bed)	6 or 10 min	6 or 10 min

Supplementary Table S2. Detailed configuration and application of the automated PET-based multi-organ segmentation pipeline. Training parameters refer to the development of the previously published pretrained PET segmentation models and not to retraining performed in the present study. The current study used the pretrained framework without cohort-specific retraining or fine-tuning. Only parameters confirmed from the source publication and the actual study workflow are reported; implementation-level settings that could not be independently verified were not inferred from generic nnU-Net defaults.

**Category**	**Parameter**	**Value / Rule**	**Description**
**Model framework**	Segmentation framework	nnU-Net version 2	Pretrained framework developed for direct multi-organ segmentation from PET images
	Network configuration	Three-dimensional full-resolution (3D-fullres)	Three-dimensional image patches are used within the nnU-Net framework
	Software implementation	Python-based implementation publicly available on GitHub	The referenced pretrained segmentation framework was used
**Training configuration of the pretrained models**	Cross-validation	Five-fold cross-validation	The pretrained models were developed using five-fold cross-validation
	Training duration	2000 epochs	Increased from the default training duration of 1000 epochs in the source framework
	Initial learning rate	(1 \times 10^{-2})	Initial learning rate reported for pretrained-model development
	Learning-rate decay	(3 \times 10^{-5}) per epoch	Learning rate was decreased at each training epoch
	Loss function	Combined Dice and cross-entropy loss	Used during development of the pretrained segmentation models
**Application in the present study**	Input modality	PET	No CT image was used as input to the segmentation model in the present study
	Segmentation input	Standard full-count PET reconstruction	The reduced-count reconstructions were not segmented independently
	Segmentation frequency	Once per patient	A single multi-organ segmentation mask was generated for each patient
	Cohort-specific model adaptation	None	No cohort-specific retraining, fine-tuning, parameter tuning, or modification of the segmentation workflow was performed

	Processing consistency	Predefined segmentation and inference workflow applied across both cohorts	The same processing rules were applied to all patients
**Application across dose levels**	Mask propagation	The mask generated from the standard full-count PET reconstruction was retained unchanged across all reduced-count reconstructions	The same mask was applied to the standard reconstruction and DRF 2, 4, 10, 20, 50, and 100
	Spatial correspondence	Intrinsic co-registration across dose levels	All full-count and reduced-count reconstructions were generated from the same original list-mode acquisition
	Dose-dependent segmentation variability	Not included	The fixed-mask design isolated count-related changes in intensity- and texture-derived radiomic features
**Quality control**	Visual assessment	Visual inspection for gross anatomical plausibility	Performed before radiomic feature extraction
	Manual correction	None	No subsequent manual modification of the generated masks was performed
**Segmentation output**	Number of anatomical regions	21	The study-defined anatomical regions are listed in Section 2.3
	Output format	Multi-organ segmentation mask	The same output mask was used for radiomic feature extraction at all dose levels

Supplementary Table S3. First-failure dose-reduction factors at the radiomics feature-family level across anatomical regions. NF indicates no failure through DRF 100, defined as maintenance of ICC ≥ 0.75 at all evaluated dose-reduction levels. Reported DRF values indicate the first evaluated dose-reduction level at which ICC decreased below 0.75. Shape features were excluded because identical PET-derived segmentation masks were applied across all dose conditions.

Anatomical region	First-order	GLCM	GLDM	GLRLM	GLSZM	NGTDM
Brain	DRF 2	DRF 2	DRF 4	DRF 2	DRF 4	DRF 2
Clavicle	DRF 2	NF	DRF 2	DRF 2	DRF 2	DRF 2
Eyes	DRF 2	NF	DRF 2	DRF 2	DRF 2	DRF 2
Femur	NF	NF	NF	NF	NF	NF
Sacral Bone	NF	NF	NF	NF	NF	NF
Ribs	NF	NF	NF	NF	NF	NF
Vertebrae	NF	NF	NF	NF	NF	NF
Hips	NF	NF	NF	NF	NF	NF
Aorta	NF	NF	NF	NF	NF	NF
Colon	NF	NF	NF	NF	NF	NF
Esophagus	NF	NF	NF	NF	NF	NF
Gallbladder	DRF 2	NF	DRF 2	DRF 2	DRF 2	DRF 2
Kidneys	NF	NF	NF	NF	NF	NF
Liver	NF	DRF 2	NF	DRF 2	DRF 2	DRF 2
Lungs	NF	NF	NF	NF	NF	NF
Adrenal Glands	DRF 2	NF	DRF 2	DRF 2	DRF 2	DRF 2
Pancreas	NF	NF	NF	NF	NF	NF
Erector Spinae	NF	NF	NF	NF	NF	NF
Stomach	NF	NF	NF	NF	NF	NF
Bladder	NF	NF	NF	NF	NF	NF
Heart	DRF 2	DRF 100	DRF 2	DRF 2	DRF 4	DRF 2

Supplementary Table S4. First-failure dose-reduction factors for selected representative radiomic features across anatomical regions. NF indicates no failure through DRF 100, defined as maintenance of ICC ≥ 0.75 at all evaluated dose-reduction levels. Reported DRF values indicate the first dose-reduction level at which ICC decreased below 0.75.

Anatomical region	Mean	Energy	Total Energy	90th Percentile	Sum Average	Joint Average	Autocorrelation
Brain	NF	NF	NF	NF	NF	NF	NF
Clavicle	NF	NF	NF	NF	NF	NF	NF
Eyes	NF	NF	NF	NF	NF	NF	NF
Femur	NF	NF	NF	NF	NF	NF	NF
Sacral Bone	NF	NF	NF	NF	NF	NF	NF
Ribs	NF	NF	NF	NF	NF	NF	NF
Vertebrae	NF	NF	NF	NF	NF	NF	NF
Hips	NF	NF	NF	NF	NF	NF	NF
Aorta	NF	DRF 2	NF	NF	NF	NF	NF
Colon	NF	NF	NF	NF	NF	NF	NF
Esophagus	NF	DRF 2	NF	NF	NF	NF	NF
Gallbladder	NF	NF	NF	NF	NF	NF	NF
Kidneys	DRF 4	NF	NF	NF	DRF 4	DRF 4	NF
Liver	DRF 4	NF	NF	DRF 10	DRF 4	DRF 4	NF
Lungs	NF	DRF 2	NF	NF	NF	NF	NF
Adrenal Glands	NF	NF	NF	NF	NF	NF	NF
Pancreas	NF	NF	NF	NF	NF	NF	NF
Erector Spinae	NF	DRF 4	NF	NF	NF	NF	NF
Stomach	NF	NF	NF	NF	NF	NF	NF
Bladder	NF	NF	NF	NF	NF	NF	NF
Heart	NF	NF	NF	NF	NF	NF	NF
